# Health literacy and fear among Iranian medical students due to COVID‐19: An observational study

**DOI:** 10.1002/brb3.2586

**Published:** 2022-04-16

**Authors:** Mohammad Pourfridoni, Moien AB Khan, Salman Daneshi, Habibe Vazirinasab, Zahra Nosrati, Milad Daneshi‐Maskooni

**Affiliations:** ^1^ Student Research Committee Jiroft University of Medical Sciences Jiroft Iran; ^2^ Health and Wellness Research Group, Department of Family Medicine, College of Medicine and Health Sciences United Arab Emirates University Al‐Ain United Arab Emirates; ^3^ Primary Care NHS Northwest London London UK; ^4^ Department of Public Health, School of Health Jiroft University of Medical Sciences Jiroft Iran; ^5^ Faculty of Medicine Kermanshah University of Medical Sciences Kermanshah Iran; ^6^ Department of Nutrition, School of Medicine Jiroft University of Medical Sciences Jiroft Iran

**Keywords:** COVID‐19, fear, health literacy, Iran, medical student, sustainable development goals

## Abstract

**Introduction:**

The coronavirus disease of the 2019 (COVID‐19) pandemic has created a sense of fear due to uncertainties in medical students’ personal and professional lives. Medical education is challenging and poses a more significant academic and emotional rigor when compared with other professional programs. With the COVID‐19 having limited treatment options, health literacy (HL) is crucial for managing and responding to the pandemic. This research aims to examine the impact of HL on COVID‐19‐associated fear among Iranian medical students.

**Methods:**

A cross‐sectional study was conducted measuring the HL and fear of COVID‐19 using validated scales. Two hundred and seventy‐eight survey responses were received and analyzed descriptively by using SPSS software inferential statistics.

**Results:**

Mean age of participants was 22.93 ± 5.427 years. The correlation between students’ total health literacy (HELIA) scores and their fear of COVID‐19 (FCV‐19S) scores was −0.279 (*p* value = .019). The FCV‐19S has a negative correlation with total HELIA in the group of females and males, single and married people, bachelor and MD students, and students living in rural areas and urban areas. The negative correlation of FCV‐19S with total HELIA in males and students who reside in rural areas was the only one that was not significant. In associate degree students, the correlation between FCV‐19S and total HELIA was positive but not significant. Education grades had a significant impact on FCV‐19S. Furthermore, the place of residence also had a significant impact on FCV‐19S. However, the gender and marital status did not significantly impact FCV‐19S.

**Conclusions:**

The present study showed that students with higher HL scores had lesser fear of COVID‐19. The key stakeholders require several positive strategies to reduce fear and improve health, and such vital policies will assist in improving the students’ health and achieving the sustainable developmental goals.

## Graphical abstract

This cross‐sectional study was conducted to investigate the effect of health literacy on fear of COVID‐19 in Iranian students. The results showed that there is a significant negative correlation between health literacy and fear of COVID‐19 among medical students. Females are more afraid of COVID‐19 than males. The key stakeholders must implement several positive strategies to reduce fear and improve health. Such key policies will assist in improving the health of the students and achieving the SDGs.

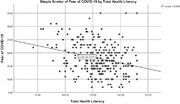

## 1 INTRODUCTION

The novel coronavirus disease 2019 (COVID‐19) pandemic is having an adverse effect due to stress on the public (Committee, 2020; Khademian et al., 2021). On February 18, 2020, Iran became the second country globally to report two deaths due to COVID‐19 after China (Rassouli et al., 2020). After the COVID‐19 outbreak in Iran, lockdown, restrictions, and other measures were placed for controlling the disease throughout the country. Despite all efforts, as of December 3, 2021, more than six million COVID‐19 cases and about 130,000 deaths due to the disease have been reported in Iran (Iran (Islamic Republic of) – WHO Coronavirus, n.d.). Such large‐scale deaths and associated negative impacts of COVID‐19 witnessed by the public have caused an aggravation of mental health problems (Shahriarirad et al., 2021).

In the current pandemic, the adverse effects had a major toll upon the students due to quarantines and other restrictions that have made their academia and life unpredictable. Furthermore, the rapid spread of COVID‐19 (Ismail et al., 2020), social isolation, frustration, worries about contracting the illness, and concerns about friends and families were associated with negative emotions and psychological distress (Lin, 2020).

Conflicting messages and associated negative publicity in the social media about the COVID‐19 pandemic have instilled many emotional reactions. Most commonly, emotional reactions like anger, guilt, fatigue, and tension are associated with COVID‐19 due to the impact of COVID‐19 on personal and professional lives (Sánchez et al., 2021). One such negative emotion that has been highly associated with the current COVID‐19 is the high amount of fear (Ahorsu et al., 2020b; Taghrir et al., 2020). There is ample evidence about the impact of fear on quality of life and mental health (Al Falasi & Ab Khan, 2020; Ghaderi et al., 2021). Medical students are often found resilient; however, they are not immune to such fears. Studies have shown that increased academic pressure and workload, financial concerns, and exposure to suffering and death have led to more stress and fear among medical students. (Dyrbye et al., 2006). The current pandemic led to the closure of universities, irregular schedules, transitioning to online medical education, cancellation of clinical placements, and fear of acquiring poor academic grades (Sandhu & de Wolf, 2020). Worries about future failure in medical careers coupled with nonacademic stressors due to health, fear of getting infected and transmitting it to others, especially their families and other personal factors, have led to fear among medical students (Alnaser et al., 2021; Marazziti et al., 2020).

Fear can have a bidirectional effect, both beneficial or detrimental to medical students’ physical or mental health. Fear can be a motivational factor leading to increased risk of perception, risk aversion leading to washing hands, social isolation, and maintaining physical distancing (Broche‐Pérez et al., 2020b). In contrast, fear can cause extreme psychological distress leading to higher levels of anxiety, depression, frustration, and inability to reason when making judgments among medical students and healthcare professionals (Shanafelt et al., 2020). Furthermore, evidence suggests fear of an epidemic can lead to a multitude of associated health problems (Zolotov et al., 2020)

Several studies have studied anxiety and mental health‐related problems among medical students in Iran (Nakhostin‐Ansari et al., 2020; Nemati et al., 2020). Though fear and anxiety are interchangeable, there is conflicting evidence regarding their origin, response pattern, and time courses (Steimer, 2002; Sullivan, 1995). Perkins et al. (2007) argue both as separable emotions, with fear having a significant impact on performance than anxiety (Perkins et al., 2007). Furthermore, such a hypothesis is strengthened by a meta‐analysis suggesting both emotions as distinct traits (Sylvers et al., 2011).

Health literacy (HL) is defined as the degree to which individuals can find, understand, and use information and services to inform health‐related decisions and actions for themselves and others (*Health literacy in Healthy People 2030 | Health.Gov*, n.d.). Evidence suggests that strengthening HL can positively impact medical students’ resilience, health behavior, and wellness (Barsell et al., 2018). HL would allow students to be more knowledgeable and informed. HL would allow students to be personally prepared and act in solidarity and achieve public health measures concerning COVID‐19 (Paakkari & Okan, 2020).

Many studies in Iran have examined the psychological association of anxiety and depression associated with COVID‐19 (Salari et al., 2020; Vahedian‐Azimi et al., 2020), and few studies have examined the relationship of COVID‐19 anxiety with HL (Mohammadkhah et al., 2021). The current work was designed to examine the variables of HL that can affect fear related to COVID‐19. The current work stemmed from the hypothesis that HL can affect fear during COVID‐19. The study aimed to identify the impact of HL on COVID −19 fear among medical students.

## 2 METHODS

### 2.1 Study population

This cross‐sectional study was conducted on the target population of medical students at Jiroft University of Medical Sciences between April 7, 2021 and May 11, 2021. The study was approved by the Research Ethics Committee of the Jiroft University of Medical Sciences, Jiroft, Iran (Ethical Approval Number: IR.JMU.REC.1400.006). The study aims and objectives were clearly explained to the participants before the study participants signed an online consent form. The study was conducted in accordance with Helsinki Declaration.

### 2.2 Data collection

Data collection was done through a web‐based questionnaire to assess the demographics, HL, and the level of fear associated with COVID‐19. The inclusion criteria were (1) being a medical student, (2) age 18 years or older, and (3) being able to understand the Persian language. All study participants met the inclusion criteria, and no one was excluded from the study. The study instrument was tested for clarity by conducting a pilot test sample on 56 participants. No changes were made to the survey instrument after pilot sampling. Later the participants were included in the final study. A convenience sampling method was used. With a 5% level of significance and 80% power to detect a minimum of 5% difference, the total sample size required to test the null hypothesis of correlation between HL and fear of COVID‐19 was calculated to be 194.

All 919 students studying at Jiroft University of Medical Sciences were invited to participate through a web‐based questionnaire. A total of 278 students responded to participate in the study out of 919 students (response rate 30.25% participants).

### 2.3 Survey measures

1. Sociodemographic variables include gender, marital status (single, married), educational grade (associate, bachelors, doctor of medicine), and place of residence (rural, urban).
2. The Health Literacy for Iranian Adults (HELIA) is a 33 items survey items instrument (Montazeri et al., 2014) that measures the five sub‐dimensions of HL, that is, access to information (six items), reading skills (four items), understanding and comprehension (seven items), appraisal (four items), and decision making (12 items). In the HELIA tool, the access reading has six items, and the participants express their agreement with the expressions in the Likert's scale (5 = quite easy, 4 = easy, 3 = neither easy nor difficult, 2 = difficult, and 1 = quite difficult). Also, in this tool, the subdomains of access, understanding, appraisal, and decision are measured with a 5‐point Likert scale (5 = always, 4 = most of the time, 3 = sometimes, 2 = rarely, and 1 = not at all). HELIA survey instrument has good reliability and validity and has been validated in several studies (Panahi et al., 2018; Zareban et al., 2016). HELIA had excellent reliability in this study with a Cronbach's alpha coefficient (CA) of 0.940.
3. Fear of COVID‐19 (FCV‐19S) is a seven‐item unidimensional, standardized, reliable, and valid tool that has been used to assess general community fear of the COVID‐19 pandemic (Ahorsu et al., 2020b). Participants express their level of agreement using a five‐point Likert scale (1 = strongly disagree, 2 = disagree, 3 = neither disagree nor agree, 4 = agree, and 5 = strongly agree). The minimum possible score for each question is 1, and the maximum is 5. By adding the scores of each question, the total score can be calculated. A higher score indicates a greater fear of the COVID‐19. The reliability and validity of the FCV‐19S have been tested in several studies and found to be a valid and reliable tool with robust psychometrics (Ahorsu et al., 2020a; Bitan et al., 2020; Pang et al., 2020; Sakib et al., 2020; Soraci et al., 2020). In our study, the reliability of the FCV‐19S measured had a CA of 0.86.

### 2.4 Data analysis

Statistical analysis was conducted using the SPSS computer package SPSS (Version 23.0) (SPSS, 2015). Data were summarized depending on the variable type, mean, standard deviation or frequency, and frequency percentage. The relationship between fear of COVID‐19 and HL and its subdomains was examined using the Pearson correlation coefficient. Independent *t*‐test and ANOVA tests were used to assess HL scores and fear of COVID‐19 in the subgroups of demographic variables. The correlation coefficient, independent *t*‐test, and ANOVA tests were two‐tailed tests. A significance level of 5% was considered significant.

## 3 RESULTS

A total of 278 students completed the survey (student participation rate = 30.25%). The mean age of participants was 22.93 ± 5.427 years. The demographic data of respondents are summarized in Table 1. HELIA had a good internal consistency and reliability with a Cronbach *α* = 0.940 and the reliability of the FCV‐19S measured had a Cronbach *α* = 0.86.

**TABLE 1:** Demographic data of participants

	Group	*n* = 278 (%)	Mean (age) ± SD
Gender	Male	86 (30.9%)	23.22 ± 5.810
	Females	192 (69.1%)	22.80 ± 5.26
Marital status	Single	236 (84.9%)	21.82 ± 3.97
	Married	42 (15.1%)	29.17 ± 7.86
Education grade	Associate	18 (6.5%)	28.44 ± 13.58
	Bachelor	197 (70.9%)	22.68 ± 4.27
	Doctor of medicine	63 (22.7%)	22.14 ± 3.71
Place of residence	Rural area	23 (8.3%)	22.39 ± 2.73
	Urban	255 (91.7%)	22.98 ± 5.61

The total HELIA score was 128.83 (18.1), and across subdomains, the score for reading was 15.68 (3.06), appraisal 15.88 (2.78), understanding 29.52 (4.29), access 23.96 (3.97), and decision 43.78 (8.56). The total FCV‐19S score was 17.99 (5.67). The total HELIA score was higher in females 128.90 (18.05) than males 128.69 (18.30), and also total FCV‐19S score was higher in females 18.34 (5.59) than males 17.23 (5.79). Although total HELIA (*p* = .927) and total FCV‐19S (*p* = .133) scores were higher in females than males, such difference was not statistically significant (Tables 2 and 3).

**TABLE 2:** Total HELIA, HELIA subdomains, and total FCV‐19S scores (*M* ± SD)

Variable	Score (*M* ± SD)
HELIA Score (total)	128.83 ± 18.1
Male	128.69 ± 18.30
Females	128.90 ± 18.05
HELIA subdomains	
Reading	15.68 ± 3.06
Appraisal	15.88 ± 2.78
Understanding	29.52 ± 4.29
Access	23.96 ± 3.97
Decision	43.78 ± 8.56
FCV‐19S score (total)	17.99 ± 5.67
Male	17.23 ± 5.79
Females	18.34 ± 5.59

**TABLE 3:** Analysis of variance for demographic variables according to total HELIA and FCV‐19S

Variable	Group	Total HELIA (mean ± SD)	FCV‐19S (mean ± SD)
Gender	Female	128.90 ± 18.05	18.34 ± 5.59
	Male	128.69 ± 18.30	17.23 ± 5.80
	*F* value	0.008	2.27
	*p* Value	.927	.133
Marital status	Single	128.31 ± 17.67	17.82 ± 5.72
	Married	131.76 ± 20.28	18.98 ± 5.33
	*F* value	1.3	1.48
	*p* Value	.256	.225
Education grade	Associate	130.67 ± 21.48	21.72 ± 6.67
	Bachelor	127.78 ± 19.02	18.14 ± 5.62
	Doctor of medicine	131.60 ± 13.43	16.49 ± 5.02
	*F* value	1.16	6.40
	*p* Value	.314	.002
Place of residence	Rural area	134.35 ± 18.80	14.96 ± 5.30
	Urban	128.34 ± 17.99	18.27 ± 5.63
	*F* value	2.34	7.37
	*p* Value	.127	.007

A significant level of .05 was considered statistically.

A one‐way ANOVA test (Table 3) was performed to determine the effect of gender, marital status, education grade, and place of residence on total HELIA and FCV‐19S. The education grades had a significant impact on FCV‐19S, *F*(2275) = 6.40, *p* = .002. Also, the place of residence had a significant impact on FCV‐19S, *F*(1276) = 7.37, *p* = .007. However, gender and marital status did not significantly impact FCV‐19S. None of the independent variables had a significant impact on total HELIA.

The correlation between the total HELIA scores and its subdomains with the total FCV‐19S score is reported in Table 4. The results show a significant negative correlation between students’ total HELIA scores and FCV‐19S score *r* = −0.279 (*p* = .019). Sub‐domains reading *r* = −0.329 (*p* < .001), appraisal *r* = −0.206 (*p* = .001), understanding *r* = −0.221 (*p* < .001), access *r* = −0.331 (*p* < .001), and decision *r* = −0.141 (*p* = .019) had a negative correlation with the FCV‐19S score.

**TABLE 4:** Correlation coefficient between total HELIA, and HELIA subdomains with FCV‐19S

	Reading	Appraisal	Understanding	Access	Decision	Total HELIA.
FCV‐19S	−0.329 (*p* < .001)	−0.206 (*p* = .001)	−0.221 (*p* < .001)	−0.331 (*p* < .001)	−0.141 (*p* = .019)	−0.279 (*p* = .019)

Correlation is significant at the .05 level.

Because age grouping was not considered during the study, only the correlation between total HELIA and FCV‐19S was reported by age in Table 5. The results showed that age was negatively correlated with total HELI.A *r* = −0.027 (*p* = .659) and positively correlated with FCV‐19S *r* = 0.061 (*p* = .315), but none of them were significant.

**TABLE 5:** Correlation coefficient between age and total health literacy and health literacy

Variable		Total HELIA	FCV‐19S
Age	Correlation	−0.027	0.061
	*p* Value	.659	.315

A significant level of .05 was considered statistically.

The correlation coefficient between total HL and fear of COVID‐19 was tested against each category. FCV‐19S had a negative correlation with total HELIA in the group of females *r* = −0.354 (*p* < .001) and males *r* = −0.122 (*p* = .261), single *r* = −0.273 (*p* < .001) and married *r* = −0.353 (*p* = .022), bachelor *r* = −0.317 (*p* < .001) and MD *r* = −0.334 (*p* = .007) students, and students living in rural areas *r* = −0.280 (*p* = .196) and urban areas *r* = −0.268 (*p* < .001). The *p* value in SPSS obtained zero for some statistical tests, but since there is no absolute zero measurement error, the *p* value is reported as *p* < .001. *p* Value <.05 is considered significant, so according to the reported *p* value, the correlation between HELIA and FCV‐19S scores is significant in females as opposed to males. In both males and females, irrespective of being single or married or living in rural or urban areas, the FCV‐19S correlated negatively with total HELIA. However, such a negative correlation of FCV‐19S with total HELIA was not significant in males and rural students. Among these, only the negative correlation of FCV‐19S with total HELIA in males and rural students was not significant. The correlation between FCV‐19S and total HELIA was positive but not significant *r* = 0.117 (*p* = .644) in associate degree students. In addition to the above, the correlation of FCV‐19S with HELIA subdomains in terms of demographic variables was reported in Table 6.

**TABLE 6:** Correlation coefficient between total health literacy and health literacy aspects with fear of COVID‐19

	Variable	Group	Reading	Appraisal	Understanding	Access	Decision	Total HELIA
FCV‐19S	Gender	Female	−0.404 (*p* < .001)	−0.274 (*p* < .001)	−0.30 (*p* < .001)	−0.417 (*p* < .001)	−0.179 (*p* = .13)	−0.354 (*p* < .001)
		Male	−0.167 (*p* = .125)	−0.027 (*p* = .808)	−0.099 (*p* = .365)	−0.153 (*p* = .159)	−0.065 (*p* = .553)	−0.122 (*p* = .261)
	Marital status	Single	−0.293 (*p* < .001)	−0.198 (*p* < .001)	−0.215 (*p* < .001)	−0.311 (*p* < .001)	−0.150 (*p* < .001)	−0.273 (*p* < .001)
		Married	−0.544 (*p* < .001)	−0.297 (*p* = .056)	−0.291 (*p* = .061)	−0.461 (*p* = .02)	−0.133 (*p* = .402)	−0.353 (*p* = .022)
	Education grade	Associate	−0.153 (*p* = .545)	0.110 (*p* = .663)	0.129 (*p* = .611)	0.042 (*p* = .869)	0.187 (*p* = .457)	0.117 (*p* = .644)
		Bachelor	−0.365 (*p* < .001)	−0.212 (*p* = .003)	−0.263 (*p* < .001)	−0.376 (*p* < .001)	−0.191 (*p* = .007)	−0.317 (*p* < .001)
		Doctor of medicine	−0.214 (*p* = .092)	−0.257 (*p* = .042)	−0.148 (*p* = .246)	−0.313 (*p* = .013)	−0.214 (*p* = .092)	−0.334 (*p* = .007)
	Place of residence	Rural area	−0.429 (*p* = .041)	−0.117 (*p* = .596)	−0.196 (*p* = .371)	−0.439 (*p* = .036)	−0.153 (*p* = .487)	−0.280 (*p* = .196)
		Urban	−0.324 (*p* < .001)	−0.213 (*p* = .001)	−0.218 (*p* < .001)	−0.308 (*p* < .001)	−0.125 (*p* = .046)	−0.268 (*p* < .001)

A significant level of .05 was considered statistically.

## 4 DISCUSSION

In our study, we aimed to assess the effect of HL on COVID‐19 associated fear among Iranian medical students. Our results highlight a COVID‐19‐associated fear among medical students. Also, there was an inverse correlation between HL scores among students and the fear of COVID‐19. Pandemic viral infections can induce widespread fear, resulting in substantial mental distress. For instance, we found significant negative correlations across all subdomains of HL, that is, access, reading, understanding, appraisal, and decision, with the fear of COVID‐19. Such a negative correlation may be due to uncertainty and lack of complete training. Multiple studies have shown that having thorough knowledge and skills can help alleviate fears (Nguyen et al., 2020; Okan et al., 2020). Terzic‐Supic et al. (2021) showed that among medical students, sufficient knowledge and fear of COVID‐19 are negatively associated (Terzic‐Supic et al., 2021). Many studies have shown a significant correlation between HL and knowledge (Bahramian et al., 2020; Chajaee et al., 2018), and hence can be inferred that a higher level of knowledge leads to higher HL. Therefore, in our study, students with sufficient knowledge were less afraid of COVID‐19. It can be inferred that higher HL in students leads to less fear of COVID‐19. Our findings are in line with a cross‐sectional study conducted by Duplaga et al., who found that during the COVID‐19 pandemic, people with higher HL scores experienced less future anxiety (Duplaga & Grysztar, 2021). In contrast, the study conducted by Shaukat et al. showed that HL did not predict fear of COVID‐19 among university students in Pakistan (Shaukat et al., 2021). One reason for this discrepancy could be the precedence of Pakistan's religious beliefs and cultural issues over health awareness (Abdullah & Zakar, 2019).

Furthermore, the current study results showed that the HL score is highest among MD students, followed by the associate and bachelor students. In our study, those with higher education grades and higher HL scores had reduced fear of COVID‐19, and our study is in line with Nguyen et al. (2020). Factors such as senior academic year, being male, and higher levels of HL may protect medical students from fear during the COVID‐19 epidemic (Nguyen et al., 2020).

Interestingly, the total HELIA score and FCV‐19S score in the married group were higher than those who were single. Also, in the married group, the negative correlation between HL and fear of COVID‐19 is significantly higher than those who are single. One of the main reasons for higher scores of fear in the married group could be the fear of infecting their loved ones and their families or losing their families due to COVID‐19 (Bernild et al., 2021; Mertens et al., 2020). Furthermore, females were more fearful than males. Our results highlight the fact that both the total HL score and the fear of COVID‐19 score was higher in females when compared to males. However, such a difference was not statistically significant. Our study findings are in line with other studies that showed women were more fearful than men. This could be related to variation in gender responses to stress and life events (Alsharawy et al., 2021; Bitan et al., 2020; Broche‐Pérez et al., 2020a; RS et al., 2020).

Current evidence suggests fear of the epidemics and pandemics may cause various health concerns (de Paiva Teixeira et al., 2020; Gaisenok, 2021; Ornell et al., 2020; Wessler et al., 2020). Such fears can impact increased mortality and morbidity along with the associated increased incidence of heart disease or diabetes (Pouwer et al., 2010; Wessler et al., 2020). In 2015, The global agenda was set by the United Nations and the members’ states to achieve 17 sustainable developmental goals (SDGs) with 169 targets by 2030 (Assembly, 2015). Our study highlights the relationship between HL and COVID‐19 fear and its impact on many SDGs and targets, in particular, SDG3 (good health and well‐being), SDG4 (quality education), SDG8 (decent work and economic growth), and SDG17 (partnerships to achieve the goal). One of the critical objectives for policymakers is to reduce and alleviate the fear during the COVID‐19 pandemic. HL is vital to such goals. The ability to read, comprehend, and act on appropriate health‐related information is vital. Individual and social resilience can be developed by improving HL. Studies have found that having a high level of HL protects against depression and anxiety, especially during the pandemic (Duplaga & Grysztar, 2021; Matterne et al., 2021; Tran et al., 2020). Detrimental effects of poor HL could be due to inadequate understanding of the COVID‐19 symptoms, infection prevention methods, difficulty finding information, misconception, and misinformation about COVID‐19 and vaccination (Biasio et al., 2021; McCaffery et al., 2020). HL is considered a key factor in public health (Kindig et al., 2004). Though the current pandemic is limited by treatment, one such improvement is to mitigate the associated fears with the COVID‐19 pandemic through education. This could be concerning the duration of quarantine, fears of infection, inadequate supplies of medications, daily provisions, finances or stigma, or need for information and services (Brooks et al., 2020; Paakkari & Okan, 2020).

The main strength of our study is that the study was conducted before the start of public vaccination against COVID‐19 in Iran because the development and implementation of public vaccination could affect the fear of COVID‐19. To the best of our knowledge, this is the first study to investigate the association between HL and fear of COVID‐19 among medical students in Iran. Furthermore, we used a valid and specific instrument that was able to gauge precisely the changes in emotions associated with HL during the current COVID pandemic.

Due to the remote implementation of this study and online data collection, the samples were not randomly selected across different geographical locations. Hence, it may not likely be a complete representative sample of the Iranian population, though fear can be associated with heightened anxiety and other mental health problems. Our study focused on a hypothesis to find the association of the fear of the COVID‐19 pandemic with HL. Hence, this resulted in a lack of ability to assess the student's mental health during the survey, which may have contributed to heightened fear. Nevertheless, as fear and anxiety can be two distinct traits of emotions (Sylvers et al., 2011), we propose a multicentric study with multiple variables associated with mental health and other emotions that could have been impacted during COVID‐19. Such a study would allow us to understand the impact of COVID‐19 on many other negative emotions that can have an impact on health.

COVID‐19 related fear and anxiety have an essential component in medical students’ physical and mental health. The findings of the study highlight the need to improve HL among medical students and the public. Key stakeholders and institutions can help to improve HL among all students during the COVID‐19 pandemic. Our study calls for global and national policies that could help reduce fear and improve the knowledge related to disease prevention and social responsibility. More important is the need for government bodies, health authorities, and health professionals to assess the citizens’ knowledge regarding the preparedness of the public for such future outbreaks in diseases.

Future research is required to investigate further how HL could be delivered effectively to improve perceptions, beliefs, and positive health outcomes.

## 5 CONCLUSION

There is a significant negative association between HL and fear of COVID‐19 among medical students. Females have higher COVID‐19‐associated fear than males. Students with higher HL scores had lower COVID‐19 fear levels than the rest. The key stakeholders must implement several positive strategies to reduce fear and improve health. Such key policies will assist in improving the health of the students and achieving the SDGs.

## AUTHOR CONTRIBUTION

M. P. conceived and designed the study. M. P. and M. D. M. participated in the organization of the activities. M. P., S. D., and Z. N. managed the activities and collected the data. H. V. and M. A. B. K. analyzed and interpreted the data. M. P., M. A. B. K., SD, H. V., Z. N., and M. D. M. drafted the article. M. P., M. A. B. K., S. D., H. V., Z. N., and M. D. M. revised the manuscript critically for important intellectual content. All authors read and approved the final article.

## CONFLICT OF INTEREST

The authors declare that there is no conflict of interest regarding the publication of this paper.

## Data Availability

The data that support the findings of this study are available from the corresponding author at reasonable request.
